# Cortisol and dehydroepiandrosterone sulfate in patients with schizophrenia spectrum disorders with respect to cognitive performance

**DOI:** 10.1016/j.cpnec.2021.100041

**Published:** 2021-03-03

**Authors:** Błażej Misiak, Patryk Piotrowski, Magdalena Chęć, Jerzy Samochowiec

**Affiliations:** a Department of Psychiatry, Division of Consultation Psychiatry and Neuroscience, Wroclaw Medical University, Wroclaw, Poland; b Department of Psychiatry, Wroclaw Medical University, Wroclaw, Poland; c Department of Clinical Psychology, Institute of Psychology, University of Szczecin, Szczecin, Poland; d Department of Psychiatry, Pomeranian Medical University, Szczecin, Poland

**Keywords:** Psychosis, Psychotic disorder, Stress, Glucocorticoids

## Abstract

Prolonged activation of the hypothalamic-pituitary-adrenal (HPA) axis associated with hypercortisolemia may lead to impairments of cognition in various populations. Dehydroepiandrosterone sulfate (DHEA-S) can protect the hippocampus from the detrimental effects of cortisol. However, this phenomenon has not been widely investigated in patients with schizophrenia spectrum disorders (SSD). Therefore, in this study, we aimed to assess the levels of cortisol, DHEA-S and cortisol/DHEA-S ratio in patients with SSD and healthy controls with respect to cognitive performance. Participants were 85 patients with SSD and 56 healthy controls, matched for age, sex and body-mass index. Cognitive performance was examined using the Repeatable Battery for the Assessment of Neuropsychological Status (RBANS). The levels of hormones were measured in fasting serum samples. The levels of morning cortisol were significantly higher in patients with SSD compared to healthy controls, even after co-varying for potential confounding factors. There were no significant between-group differences in the levels of DHEA-S and cortisol/DHEA-S ratio. Higher levels of cortisol and greater cortisol/DHEA-S ratio were related to significantly lower RBANS scores of delayed memory in patients with SSD, but not in healthy controls after controlling for the effects of age, sex, BMI, the number of education years, cigarette smoking status and the dosage of antipsychotics. Our findings imply that elevated cortisol levels may contribute to impairments of memory processes in patients with SSD. However, longitudinal studies are needed to confirm causal associations.

## Highlights

• Patients with schizophrenia show increased morning cortisol levels.
• Our findings do not indicate altered levels of DHEA-S in schizophrenia.
• Higher cortisol levels might be related to impaired delayed memory in schizophrenia.

## 1 Introduction

Schizophrenia is a chronic mental disorder that leads to considerable disability [1]. Typical manifestation of schizophrenia includes positive symptoms (hallucinations and delusions), negative symptoms (blunted affect, alogia, avolition, anhedonia and social withdrawal), affective symptoms and cognitive impairment. Apart from a clear role of genetic factors, a number of environmental exposures have been identified to increase a risk of schizophrenia. For instance, it has been reported that stressful life events at various life periods play an important role in shaping a risk of schizophrenia and triggering psychotic relapse [2,3]. Moreover, there is evidence that patients with schizophrenia often approach maladaptive coping strategies [4,5] and show lower levels of resilience [6].

Patients with schizophrenia show impaired multisystemic responses to stress [7]. These impairments are mainly associated with dysregulated activity of the hypothalamic-pituitary-adrenal (HPA) axis, and manifest in increased morning cortisol levels [8], blunted cortisol awakening response (CAR) [9] and reduced cortisol response to stress [10,11]. Moreover, altered methylation of genes encoding the HPA axis proteins has been found in patients with schizophrenia [12,13]. Higher diurnal cortisol levels and blunted CAR have been associated with reduced hippocampal volumes in patients with first-episode psychosis [14,15]. This observation would explain the association between the HPA axis functioning and cognitive performance in patients with schizophrenia. Indeed, it has been reported that higher cortisol levels are related to worse performance of executive [16] and memory functions [[17], [18], [19]].

However, there are also other hormones involved in the regulation of the HPA axis activity. These hormones are represented by dehydroepiandrosterone (DHEA) sulfate (DHEA-S) that is released from the adrenal gland. Notably, DHEA-S enters the brain and can be used as a local substrate for synthesis of neurosteroids. It can also modulate dopaminergic, glutamatergic and GABAergic neurotransmission [20]. Moreover, DHEA-S is involved in biological responses to stress and exerts anti-glucocorticoid activity by protecting the hippocampus from the effects of corticosterone [21]. A recent meta-analysis revealed that elevated DHEA-S levels might appear in patients with first-episode psychosis [22]. There is also some evidence from non-psychiatric populations that higher levels of DHEA and DHEA-S might be associated with better cognitive performance [23].

However, studies investigating the effects of the HPA axis activity in patients with schizophrenia rarely include the measurement of DHEA-S levels. The DHEA antagonism to cortisol in the brain indicate that measurements limited to cortisol levels might be insufficient to assess hypercortisolemia. Therefore, a more precise method to assess “functional hypercortisolemia” might be to calculate the cortisol/DHEA ratio [24,25]. Ji et al. [26] found elevated levels of DHEA and decreased levels of cortisol/DHEA ratio in patients with schizophrenia. Additionally, the authors demonstrated a significant negative correlation of cortisol/DHEA ratios with hippocampal and prefrontal cortex volumes. However, this study did not include any measures of cognitive performance. Therefore, in this study we aimed to determine the levels of cortisol, DHEA-S and cortisol/DHEA-S ratio in patients with schizophrenia spectrum disorders and healthy controls. We hypothesized that the levels of cortisol and DHEA-S as well as cortisol/DHEA-S ratio are elevated in patients with schizophrenia spectrum disorders. Moreover, we established the hypothesis that higher levels of cortisol and cortisol/DHEA-S ratio as well as lower levels of DHEA-S are associated with worse cognitive performance in this group of patients.

## 2 Material and methods

### 2.1 Participants and procedures

In the present study, 85 inpatients with schizophrenia spectrum disorders and 56 healthy controls were recruited at two university hospitals in Wroclaw and Szczecin (Poland). Patients met the DSM-IV criteria for the following diagnoses: schizophrenia (n ​= ​59), schizoaffective disorder (n ​= ​5), schizophreniform disorder (n ​= ​7), brief psychotic disorder (n ​= ​13) and delusional disorder (n ​= ​1). All diagnoses were confirmed using the Operational Criteria for Psychotic Illness (OPCRIT) checklist [27]. Both groups of participants were matched for age, sex and body-mass index (BMI). Healthy controls had never received psychiatric diagnosis or treatment, and reported no family members affected by psychotic and affective disorders in first- and second-degree relatives. They were enrolled through advertisements.

The following measures of psychopathological manifestation were administered: 1) the Positive and Negative Syndrome Scale (PANSS) [28]; 2) the Montgomery-Asberg Depression Rating Scale (MADRS) [29], 3) the Young Mania Rating Scale (YMRS) [30] and 4) the Social and Occupational Functioning Assessment Scale (SOFAS) [31]. Cognitive performance was examined using the Repeatable Battery for the Assessment of Neuropsychological Status (RBANS) [32]. The RBANS is composed of 12 tasks measuring 5 domains of cognitive performance: 1) immediate memory (list learning and story memory); 2) visuospatial/constructional functions (figure copy and line orientation); 3) language (picture naming and semantic fluency) 4) attention (digit span and coding) and 5) delayed memory (list recall, list recognition, story memory and figure recall).

The levels of cortisol and DHEA-S were determined in fasting blood samples collected between 7 and 9 a.m. (total volume of collected blood was 15 ​ml). Electrochemiluminescence analysis was performed to measure the levels of hormones (Cobas e411 analyzer, Roche). The detection range was 0.1–1000.0 ​μg/dl for DHEA-S and 1.5–1750.0 ​nmol/l for cortisol. All participants signed written informed consent and the protocol of this study was approved by the Ethics Committee at Wroclaw Medical University (Wroclaw, Poland).

### 2.2 Data analysis

Results of this study were analyzed using the Statistical Package for Social Sciences, version 20 (SPSS Inc., Chicago, Illinois, USA). Differences in the distribution of categorical variables were assessed using the chi-squared test. Due to non-normal distribution, the Mann-Whitney *U* test was applied to examine differences in continuous variables. The Spearman rank correlation coefficients were used to test correlations between the levels of hormones and cognitive performance. In case of significant bivariate correlations, linear regression analyses were performed. The level of cognitive performance was included as the dependent variable. Age, sex, BMI, the number of education years, chlorpromazine equivalent dosage (CPZeq), cigarette smoking and the levels of hormones were used as independent variables. Linear regression analyses were performed separately for patients and healthy controls. Similarly, in case of significant differences in the levels of hormones, the analysis of co-variance (ANCOVA) was carried out. Age, sex, BMI, the number of education years, CPZeq and cigarette smoking status were included as co-variates. Results of data analysis were interpreted as significant if the p-value was less than 0.05.

## 3 Results

**Fig. 1:**
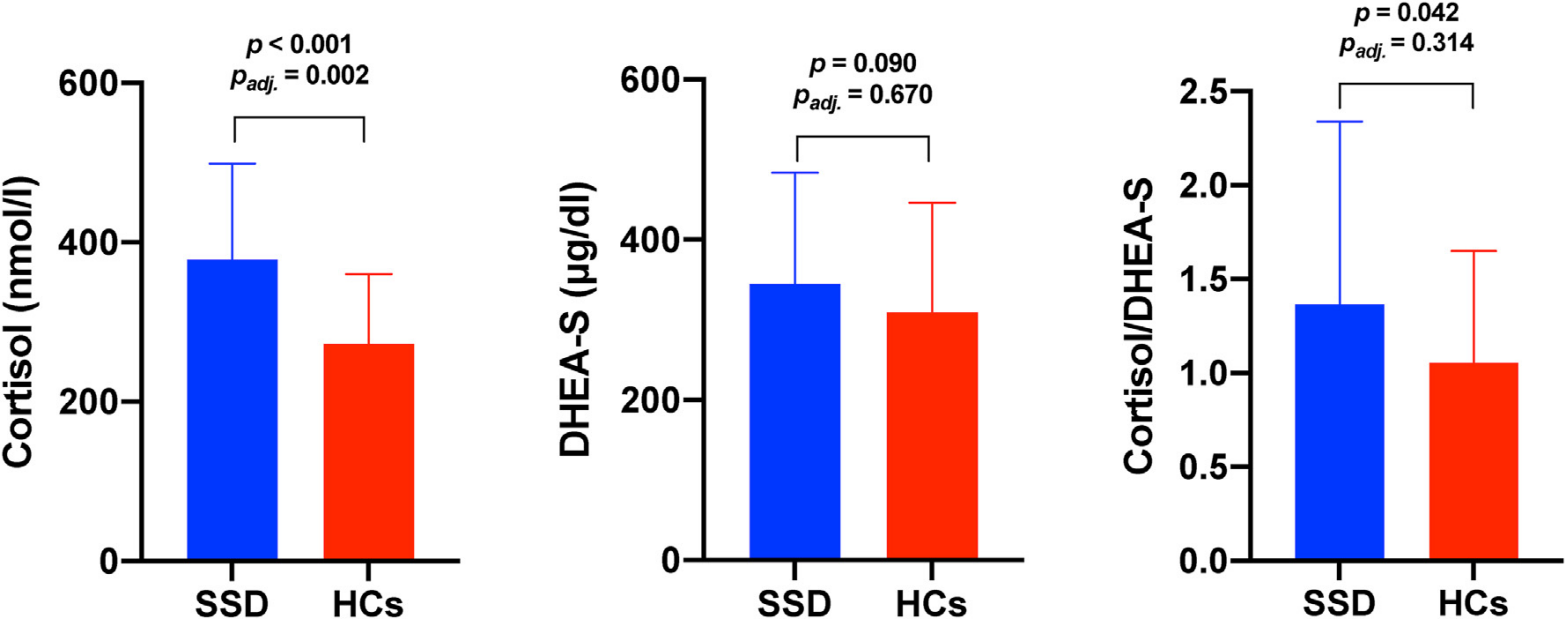
The levels of cortisol, DHEA-S and cortisol/DHEA-S ratio in patients with schizophrenia spectrum disorders (SSD) and healthy controls (HCs).

The comparison of patients with schizophrenia spectrum disorders and healthy controls is shown in Table 1. There were no significant differences in age, sex and BMI. As expected, the level of cognitive performance on all domains was significantly lower in the group of patients compared to healthy controls. Bivariate comparisons revealed that the levels of cortisol and cortisol/DHEA-S ratio were significantly higher in patients with schizophrenia-spectrum disorders than in healthy controls (Fig. 1). Differences in the levels of cortisol remained significant (F ​= ​10.598, p ​= ​0.002) after adjustment for age, sex, the number of education years, BMI, cigarette smoking and CPZeq. However, differences in the levels of DHEA-S (F ​= ​0.183, p ​= ​0.670) and cortisol/DHEA-S ratio (F ​= ​1.023, p ​= ​0.314) were not significant after co-varying for potential confounding factors.

**Table 1:** General characteristics of patients and healthy controls.

	SSD	HCs	p
**Age, years**	37.1 ​± ​13.5	32.5 ​± ​8.2	0.120
**Sex, males**	52.9%	39.3%	0.124
**Education, years**	13.1 ​± ​2.8	15.8 ​± ​2.5	<0.001
**BMI, kg/m**^**2**^	25.1 ​± ​4.7	23.8 ​± ​3.5	0.074
**Cigarette smoking, yes**	46.2%	8.9%	<0.001
**SOFAS**	45.2 ​± ​16.3	96.3 ​± ​5.5	<0.001
**RBANS – immediate memory**	37.9 ​± ​11.0	51.9 ​± ​6.0	<0.001
**RBANS – visuospatial/constructional abilities**	32.2 ​± ​7.4	38.1 ​± ​2.2	<0.001
**RBANS – language**	26.5 ​± ​6.6	33.7 ​± ​6.5	<0.001
**RBANS – attention**	44.5 ​± ​15.2	68.9 ​± ​8.9	<0.001
**RBANS – delayed memory**	42.8 ​± ​10.5	56.0 ​± ​4.5	<0.001
**PANSS-P**	14.1 ​± ​5.2	–	–
**PANSS-N**	20.9 ​± ​9.3	–	–
**MADRS**	8.1 ​± ​8.1	–	–
**YMRS**	2.1 ​± ​5.0	–	–
**CPZeq, mg/day**	380.6 ​± ​211.6	–	–

Abbreviations: BMI – body-mass index; CPZeq – chlorpromazine equivalent dosage; HCs – healthy controls; MADRS – the Montgomery-Asberg Depression Rating Scale; PANSS-N – the Positive and Negative Syndrome Scale (subscale of negative symptoms); PANSS-P – the Positive and Negative Syndrome Scale (subscale of positive symptoms); RBANS – the Repeatable Battery for the Assessment of Neuropsychological Status; SOFAS – the Social and Occupational Functioning Assessment Scale; SSD – schizophrenia spectrum disorders; YMRS – the Young Mania Rating Scale.

Correlations between the levels of hormones and cognitive performance are presented in Table 2. There were significant negative correlations of cortisol levels with performance of attention and delayed memory in the group of patients. Higher cortisol/DHEA-S ratio was associated with lower scores of delayed memory in patients with schizophrenia spectrum disorders. Correlations of cortisol levels and cortisol/DHEA-S ratio with the levels of delayed memory were significant in linear regression analyses after controlling for the effects of age, sex, BMI, cigarette smoking, the number of education years and CPZeq. No significant correlations between the levels of hormones and cognitive performance were found in healthy controls.

**Table 2:** Correlations between the levels of hormones and cognitive performance.

	Cortisol		DHEA-S		Cortisol/DHEA-S	
	Psychosis	Controls	Psychosis	Controls	Psychosis	Controls
RBANS – immediate memory	r ​= ​−0.157,	r ​= ​0.069,	r ​= ​−0.030,	r ​= ​0.127,	r ​= ​−0.102,	r ​= ​−0.119,
	p ​= ​0.223	p ​= ​0.631	p ​= ​0.814	p ​= ​0.376	p ​= ​0.430	p ​= ​0.405
RBANS – visuospatial/	r ​= ​−0.239,	r ​= ​0.033,	r ​= ​0.061,	r ​= ​0.029,	r ​= ​−0.212,	r ​= ​−0.039,
constructional abilities	p ​= ​0.062	p ​= ​0.816	p ​= ​0.638	p ​= ​0.837	p ​= ​0.098	p ​= ​0.788
RBANS – language	r ​= ​−0.039,	r ​= ​−0.012,	r ​= ​0.031,	r ​= ​0.079,	r ​= ​−0.061,	r ​= ​−0.185,
	p ​= ​0.766	p ​= ​0.934	p ​= ​0.813	p ​= ​0.583	p ​= ​0.638	p ​= ​0.195
RBANS - attention	r ​= ​−0.255,	r ​= ​0.010,	r ​= ​0.034,	r ​= ​−0.024,	r ​= ​−0.205,	r ​= ​−0.009,
	p ​= ​0.046^a^	p ​= ​0.944	p ​= ​0.791	p ​= ​0.866	p ​= ​0.110	p ​= ​0.950
RBANS – delayed memory	r ​= ​−0.318,	r ​= ​−0.015,	r ​= ​0.087,	r ​= ​0.205,	r ​= ​−0.256,	r ​= ​−0.180,
	p ​= ​0.012^b^	p ​= ​0.917	p ​= ​0.503	p ​= ​0.149	p ​= ​0.045^c^	p ​= ​0.206

a Linear regression analysis: age (B ​= ​−0.686, t ​= ​−4.835, p ​< ​0.001), sex (B ​= ​2.070, t ​= ​0.608, p ​= ​0.546), education years (B ​= ​2.217, t ​= ​3.504, p ​= ​0.001), BMI (B ​= ​−0.403, t ​= ​−1.039, p ​= ​0.303), cigarette smoking (B ​= ​2.088, t ​= ​0.603, p ​= ​0.549), CPZeq (B ​= ​−0.006, t ​= ​−0.754, p ​= ​0.454), cortisol (B ​= ​−0.019, t ​= ​−1.306, p ​= ​0.197).

b Linear regression analysis: age (B ​= ​−0.152, t ​= ​−1.329, p ​= ​0.189), sex (B ​= ​−1.776, t ​= ​−0.647, p ​= ​0.521), education years (B ​= ​0.826, t ​= ​1.617, p ​= ​0.112), BMI (B ​= ​−0.239, t ​= ​−0.763, p ​= ​0.449), cigarette smoking (B ​= ​−3.636, t ​= ​−1.302, p ​= ​0.199), CPZeq (B ​= ​−0.002, t ​= ​−0.376, p ​= ​0.709), cortisol (B ​= ​−1.345, t ​= ​−2.476, p ​= ​0.046).

c Linear regression analysis: age (B ​= ​−0.165, t ​= ​−1.294, p ​= ​0.201), sex (B ​= ​−1.790, t ​= ​−0.552, p ​= ​0.584), education years (B ​= ​0.736, t ​= ​1.415, p ​= ​0.163), BMI (B ​= ​−0.344, t ​= ​−1.094, p ​= ​0.279), cigarette smoking (B ​= ​−3.307, t ​= ​−1.158, p ​= ​0.252), CPZeq (B ​= ​−0.003, t ​= ​−0.413, p ​= ​0.681), cortisol/DHEA-S ratio (B ​= ​−0.326, t ​= ​−2.112, p ​= ​0.049).

## 4 Discussion

The present study demonstrated significantly higher levels of cortisol in patients with schizophrenia spectrum disorders compared to healthy controls. There were no significant between-group differences in DHEA-S levels and cortisol/DHEA-S after controlling for the effects of potential confounding factors. Moreover, we found that higher levels of cortisol and greater cortisol/DHEA-S ratio are associated with worse performance of delayed memory after controlling for potential confounding factors in the group of patients but not in healthy controls.

It has clearly been shown that the hippocampus is one of key structures responsible for learning processes and consolidation of various information sources into the long-term memory storage [33]. It contains a high density of glucocorticoid receptors, regulating the activity of the HPA axis and enabling to classify specific situations as threatening or not, according to the information stored in a long-term memory [34,35]. There is compelling evidence that acute administration of glucocorticoids leads to long-term impairment of memory retrieval [36]. However, stronger release of cortisol after acute stress has been associated with a greater deactivation of the hippocampus, indicating that this structure can inhibit the activity of the HPA axis [36,37]. In turn, prolonged exposure to cortisol may produce morphological and molecular alterations, reduced neurogenesis and impaired synaptic plasticity in the hippocampus [38].

A recent meta-analysis of post mortem studies demonstrated reduced volume and neuron numbers in multiple subfields of the left, but not right, hippocampus of individuals with schizophrenia [39]. Bilateral neuron size was also decreased in all analyzed subfields. Importantly, age and illness duration did not explain heterogeneity of effect size estimates. A meta-analysis of neuroimaging studies revealed similar findings [40]. Moreover, higher diurnal cortisol levels and blunted CAR have been associated with reduced hippocampal volumes in patients with first-episode psychosis [14,15]. These observations and our findings are also in line with previous studies showing that higher cortisol levels are associated with worse performance of memory functions in patients with schizophrenia [[17], [18], [19]]. A lack of significant correlations between cortisol levels and cognitive performance in healthy controls might be explained by the fact that cortisol levels were significantly lower in this group of participants. Therefore, detrimental effects of hypercortisolemia on cognition could not be observed.

Importantly, we did not observe any significant alterations of DHEA-S levels in patients with schizophrenia spectrum disorders. These findings are in contrast with those obtained by Ji et al. [26], who found elevated levels of DHEA and decreased levels of cortisol/DHEA ratio in patients with schizophrenia. However, our sample was based on inpatients, while Ji et al. [26] recruited outpatients. This is a relatively important difference as the levels of DHEA and DHEA-S might differ with respect to phase of illness [22]. Similarly, timing of biological material sampling is an important factor, as the HPA axis activity might be elevated at stress exposure and decreases as time passes [41]. There are also some other studies that reported no significant differences in the levels of DHEA-S between patients with schizophrenia and healthy controls [[42], [43], [44]]. As similar to our findings, a lack of significant correlations between the levels of DHEA-S and cognitive performance has also been reported in patients with schizophrenia [45] and non-clinical populations [23]. Also, available evidence does not support the use of DHEA to improve cognition in non-demented middle-aged or elderly population [46].

The present study is characterized by certain methodological limitations that should be discussed. First, our sample was not large and there was some diagnostic heterogeneity. Second, this study was based on single measurements of cortisol and DHEA-S levels. Another limitation is that medication effects cannot be excluded as we recorded only the dosage of antipsychotics. Additionally, we did not include any neuroimaging measures, and thus we cannot attribute our observations to specific neural substrates. Finally, a lack of longitudinal design does not allow to conclude regarding causal associations.

In summary, results of this study indicate that inpatients with schizophrenia spectrum disorders show elevated morning cortisol levels, even when various potential confounding factors are taken into consideration. Higher levels of cortisol and greater cortisol/DHEA-S ratio might contribute to impairments of delayed memory in this clinical population. Targeting the HPA axis dysfunction in people with schizophrenia spectrum disorders might provide grounds for novel interventions that aim to improve cognition. However, longitudinal studies are still needed to confirm causal associations between dysregulated HPA axis activity and cognitive impairment in schizophrenia.

## Funding

This study was funded by the Iuventus Plus grant awarded by the Ministry of Science and Higher Education (IP2015 052474).

## Declaration of competing interest

None to declare.
